# Functional Assessment of Human Coding Mutations Affecting Skin Pigmentation Using Zebrafish

**DOI:** 10.1371/journal.pone.0047398

**Published:** 2012-10-10

**Authors:** Zurab R. Tsetskhladze, Victor A. Canfield, Khai C. Ang, Steven M. Wentzel, Katherine P. Reid, Arthur S. Berg, Stephen L. Johnson, Koichi Kawakami, Keith C. Cheng

**Affiliations:** 1 Jake Gittlen Cancer Research Foundation, Penn State Hershey College of Medicine, Hershey, Pennsylvania, United States of America; 2 Department of Pharmacology, Penn State Hershey College of Medicine, Hershey, Pennsylvania, United States of America; 3 Department of Public Health Sciences, Penn State Hershey College of Medicine, Hershey, Pennsylvania, United States of America; 4 Department of Genetics, Washington University Medical School, St. Louis, Missouri, United States of America; 5 Division of Molecular and Developmental Biology, National Institute of Genetics, Department of Genetics, Graduate University for Advanced Studies (Sokendai), Mishima, Shizuoka, Japan; 6 Division of Experimental Pathology, Department of Pathology, Penn State Hershey College of Medicine, Hershey, Pennsylvania, United States of America; Texas A&M University, United States of America

## Abstract

A major challenge in personalized medicine is the lack of a standard way to define the functional significance of the numerous nonsynonymous, single nucleotide coding variants that are present in each human individual. To begin to address this problem, we have used pigmentation as a model polygenic trait, three common human polymorphisms thought to influence pigmentation, and the zebrafish as a model system. The approach is based on the rescue of embryonic zebrafish mutant phenotypes by “humanized” zebrafish orthologous mRNA. Two hypomorphic polymorphisms, *L374F* in *SLC45A2,* and *A111T* in *SLC24A5,* have been linked to lighter skin color in Europeans. The phenotypic effect of a second coding polymorphism in *SLC45A2*, *E272K,* is unclear. None of these polymorphisms had been tested in the context of a model organism. We have confirmed that zebrafish *albino* fish are mutant in *slc45a2*; wild-type *slc45a2* mRNA rescued the *albino* mutant phenotype. Introduction of the *L374F* polymorphism into *albino* or the *A111T* polymorphism into *slc24a5* (*golden*) abolished mRNA rescue of the respective mutant phenotypes, consistent with their known contributions to European skin color. In contrast, the *E272K* polymorphism had no effect on phenotypic rescue. The experimental conclusion that *E272K* is unlikely to affect pigmentation is consistent with a lack of correlation between this polymorphism and quantitatively measured skin color in 59 East Asian humans. A survey of mutations causing human oculocutaneous albinism yielded 257 missense mutations, 82% of which are theoretically testable in zebrafish. The developed approach may be extended to other model systems and may potentially contribute to our understanding the functional relationships between DNA sequence variation, human biology, and disease.

## Introduction

Studies of individual genome sequences have revealed that every human possesses several thousand sequence variants that alter coding sequence, most of which are rare at the population level [1], [2]. Published computational approaches to identifying functional effects of amino acid variation exhibit poor concordance [2]. These observations indicate a need for *in vivo* approaches to the experimental assessment of the functional significance of individual mutations.

To begin to address this need, we have chosen the zebrafish as a model system because of the high degree of conservation of genes between fish and humans, the ability to score mutant phenotypes in the context of the organismal and tissue structure of the whole animal (especially embryonic phenotypes), and the growing ease of inhibiting the function of specific genes in zebrafish by antisense methods [3] or by mutation [4], [5]. The approach presented and referred to here as “Humanized Zebrafish Orthologous Rescue” (HuZOR) is based on the ability to rescue embryonic zebrafish mutant phenotypes by microinjection of mRNA into fertilized eggs.

We have chosen pigmentation as a phenotype that is readily scored in humans and model systems. Rare null or strongly hypomorphic mutations in a number of pigmentation genes cause oculocutaneous albinism, while coding polymorphisms in at least two pigmentation genes have played a role in the evolution of human skin color. Here we focus on *SLC45A2* and *SLC24A5* [6], [7]. *SLC45A2* (also called *AIM1* and *MATP*) was initially identified as a pigmentation gene through the positional cloning of the genes mutated in the hypopigmented medaka *b* [8] and mouse *underwhite* (*uw*) mutants [9]. In humans, coding mutations [10], splice junction mutations [9], deletions and insertions [11] cause oculocutaneous albinism type 4 (OCA4). Two common coding polymorphisms in *SLC45A2* are also known. The *L374F* (rs16891982) polymorphism changes the ancestral leucine to a phenylalanine at amino acid 374 (we use the convention in which the evolutionarily ancestral amino acid, rather than that of the reference genome, is given first). This derived allele is nearly fixed in populations of European ancestry, and has been associated with variation in normal human skin, hair, and eye color between and within populations [12], [13], [14]. *L374F* has also been associated with increased risk of both basal and squamous cell carcinoma [15]. A second polymorphism, *E272K* (rs26722), which changes the ancestral glutamic acid to a lysine at amino acid 272, is found in East Asian populations, with derived allele frequencies of about 0.4, compared with European and African frequencies of ≤0.05 [12], [16]. The association of this variant by Graf et al. with darker pigmentation [12], may be explained by linkage disequilibrium [17], but experimental evidence has been lacking.

The positional cloning of the zebrafish *golden* gene led to identification of *slc24a5* as a previously unidentified pigmentation gene, while analysis of admixed populations established that the human *A111T* polymorphism (rs1426654) in *SLC24A5* accounts for 25–38% of the difference in skin color between West Africans and Europeans [7]. Nearly all people of European ancestry are homozygous for the derived allele. Targeted inactivation of *Slc24a5* in mouse causes mild ocular hypopigmentation [18].

Here we describe the development of an experimental approach to assessing the functional significance of specific human mutations affecting coding sequence. There are examples of rescue of antisense knock-down (morphant) phenotypes using human mRNA [19], but as described below, this is not always possible. In our approach, zebrafish mutants in orthologous genes are used to assess the effects of human nonsynonymous coding mutations on gene function. Orthologous zebrafish cDNAs are engineered to contain codons corresponding to the human polymorphic variants. The approach relies upon phenotypic rescue of mutant phenotypes by mRNA injection [20], which is often used in zebrafish genetics [21]. For *SLC45A2* we used *albino^nk1^*, which was cloned here, and for *SLC24A5*, we used *golden^b1^* as the recipient strain. These mutants, and the quantitative study of skin color in an East Asian human population sample, allowed us to demonstrate the feasibility of using zebrafish to assess the impact of human mutations affecting coding sequence.

## Results

### The Zebrafish *albino* Gene is *slc45a2*

**Figure 1:**
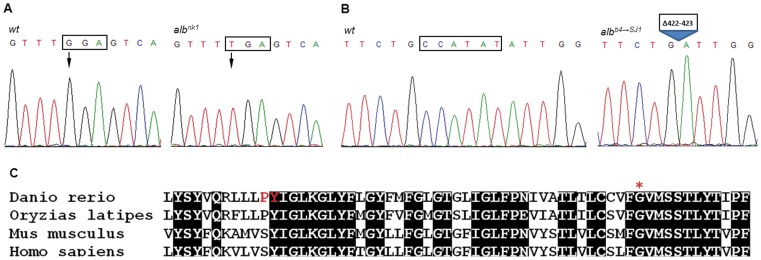
Sequence analysis of the *albino* gene. (A and B) Sequence traces of genomic DNA show (A) G→T mutation in *slc45a2^nk1^* (boxed) and (B) 2aa (6 nt) allele *slc45a2* ^Δ*422–423*^ deletion (boxed) (C) Alignment of *slc45a2* from various vertebrate species showing disruption of conserved sequences in both the two amino acid deletion (PY red) *alb^b4→SJ1^* and the nonsense mutation GGA(Gly461)>UGA(stop) (red asterisk) of *alb^nk1^* zebrafish albino mutants in exon 6. Identical sequences are shown in black.

**Figure 2:**
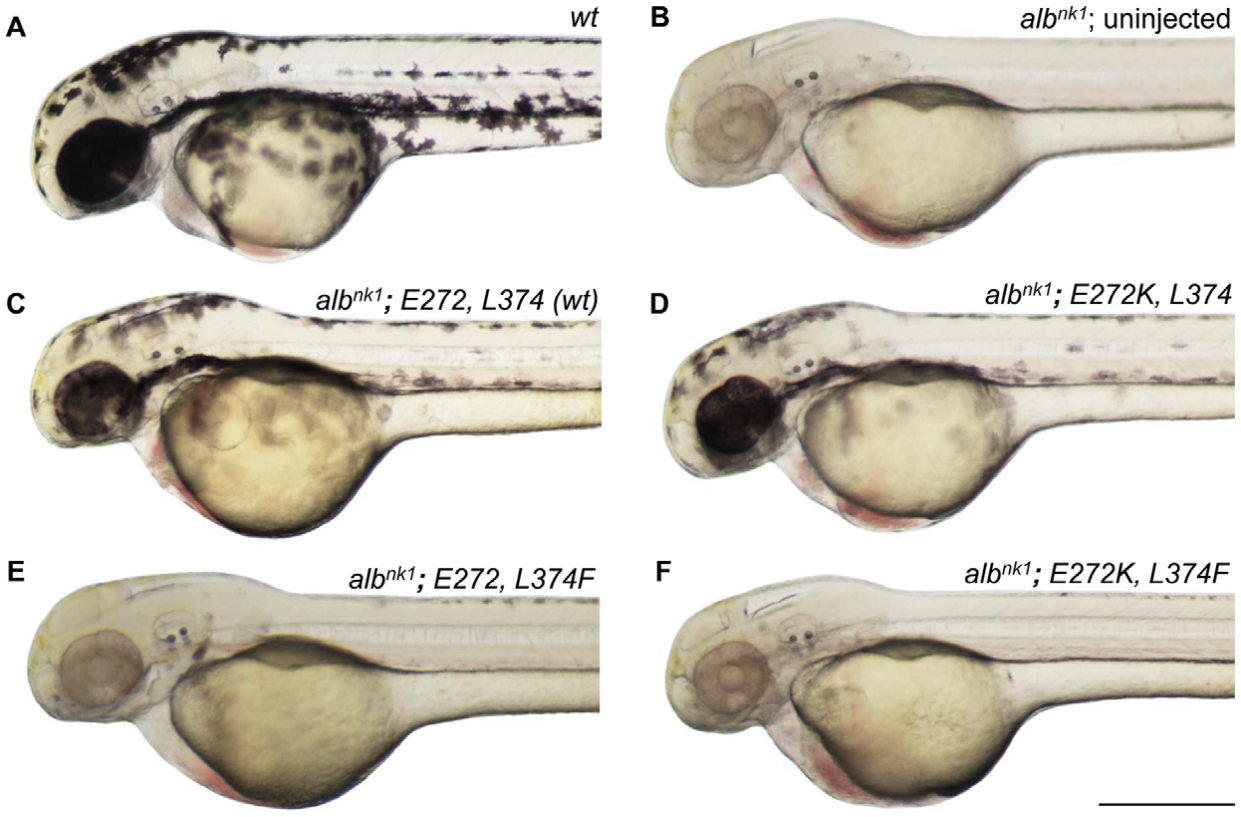
Effect of human coding polymorphisms on zebrafish mRNA rescue of the *albino* phenotype. Lateral views of 48-hpf (A) wild-type zebrafish larva (B) un-injected *alb^nk1^* zebrafish larva (C-F) *alb^nk1^* zebrafish larva injected with mRNA (500 pg), coding for indicated variants of zebrafish *slc45a2*, named according to positions of human variation (human 272 equivalent to zebrafish 303; human 374 equivalent to zebrafish 403). (C) wild-type; (D) *E272K* mutant; (E) *L374F* mutant; (F) *E272K/L374F* double mutant. Note that mRNA rescue in zebrafish does not occur in every cell, which is thought to be due to unequal distribution of the mRNA in the cytoplasm of the originally injected eggs, resulting in unequal distribution of the mRNA among different cells of the embryo. The results shown are typical of a majority of injected embryos in each case; each of embryo in the majority populations contains cells as pigmented as the ones evident in this figure. Scale bar 400, µm.

To test the functional impact of human coding polymorphisms on *SLC45A2* gene function, we first needed a zebrafish mutant for the orthologous gene. The common zebrafish *albino* mutant (*alb^b4^*) causes a loss of melanin pigmentation, without any apparent reduction in xanthophore or leucophore pigmentation [22]. We noted that the map position of this mutation, LG21 at 52 cM [23] is coincident with the location of *slc45a2*. Knockdown of *slc45a2* caused an *albino* phenotype (Figure S1). We characterized three *albino* alleles, an *alb^b4^* allele from the Zebrafish International Resource Center at U Oregon (ZIRC), the *alb^nk1^* from the Kawakami lab in Japan and the *alb^b4→SJ1^* allele (derived from *alb^b4^* allele) from the Johnson lab at Washington University. Sequencing of *alb^b4^* revealed a large insert (ZRT and KC, unpublished data). The *alb^nk1^* allele of *slc45a2* revealed a nonsense mutation in exon 6 that truncates the protein at amino acid (aa) 461 [GGA (Gly)>UGA] to about 83% of its normal size (Figure 1A). The *alb^b4→SJ1^* allele of *slc45a2* possesses a 6 nt deletion, also located in exon 6, that results in a 2aa deletion (Δ422–423 = PY**)** (Figure 1B). Polypeptide alignments revealed that the insertion and the deletion lies within a segment of evolutionarily conserved amino acids (Figure 1C). No other nucleotide differences predicted to change amino acids were identified in either mutant. To verify that these specific mutations were causative, we employed rescue using synthetic mRNA [20], [21]. The embryonic hypopigmentation phenotypes of *alb^nk1^* and *alb^b4→SJ1^* were rescued by the injection of 500 pg *wild-type* (*wt*) zebrafish *slc45a2 mRNA* (Figure 2C). Injection of mRNA containing only the 6 nt deletion corresponding to the *alb^b4→SJ1^* allele showed no rescue in *alb^nk1^* (data not shown), confirming the ability of this deletion to reduce protein activity. Taken together, these experiments confirm that mutations in *slc45a2* are responsible for the hypopigmentation phenotype of *albino*, and establish the feasibility of testing *slc45a2* polymorphisms by mRNA rescue of *albino* mutants.

### Rescue using Human mRNA and mRNA Rescue of Morphants may not Always be Possible

Direct testing of human coding mutations can theoretically be done by injecting the human mRNA into zebrafish mutants [7] or into morphants for the orthologous gene [19], [24]. However, when we attempted to compare rescue of pigmentation in *alb^nk1^* using human *SLC45A2* mRNAs carrying ancestral and *L374F* alleles, we found that doses above 1000 pg produced defects in embryonic development, but neither allele rescued pigmentation at doses up to 1400 pg (Figure S2B, C). This result contrasts strongly with our observations using *wt* zebrafish *slc45a2* mRNA, which rescued pigmentation even at high doses that cause similarly substantial developmental defects (Figure S2A). The basis for rescue by zebrafish but not human mRNA in this case is uncertain. The potential presence of species-specific effects and the presence of multiple amino acid differences between species, together suggest that it may be preferable to engineer zebrafish sequences that correspond to human variants rather than to use the human sequences directly.

Knockdowns using antisense morpholino oligonucleotides (MOs) are a standard technique widely used in zebrafish [3]. MOs targeted against *slc45a2* causes an *albino* phenotype when injected into *wt* zebrafish (Figure S1). We used a 5′ UTR-directed MO targeted to the endogenous *slc45a2*. We designed the MO to not overlap with injected mRNA (which substitutes a β-globin leader for the endogenous sequence) to allow us to test rescue by specific alleles. However, co-injection of morpholino with mRNA produced embryonic defects (Figure S3) far exceeding those seen when either morpholino or mRNA was injected singly. Morphological defects associated with morpholino injection obscure the potential rescue of morphant phenotypes for *slc45a2* (Figure S3C). Similar results were obtained for *slc24a5* (Figure S4). The use of mRNA rescue of morphants is feasible for at least some genes [3], [25], but to test human alleles for *SLC24A5* and *SLC45A2*, we needed an alternative to using morphants in allele-specific rescue experiments.

### Testing Humanized Zebrafish *L374F* and *E272K* mRNA Alleles of *SLC45A2* in *albino*

Based on the above results, we decided to test an alternative approach to assessing human mutations that is based on the successful rescue of zebrafish mutants by injection of mRNA. Site-directed mutagenesis was used to create orthologous mutations in zebrafish cDNAs, which were then used to generate mRNAs for rescue experiments. This approach, referred to here as Humanized Zebrafish Orthologous Rescue (HuZOR), was used to test three human coding variants.

To analyze human *SLC45A2* polymorphisms using HuZOR, we began with the zebrafish *albino* mutant, which was shown above to be mutant in the orthologous zebrafish *slc45a2* gene. We then generated each possible combination of zebrafish *E272/E272K* and *L374/L374F* (named using human numbering; corresponding positions in zebrafish are 303 and 403, respectively). The zebrafish *wt* corresponds to *E272/L374*, and coincides with the ancestral human state. Zebrafish *slc45a2^wt^* and *slc45a2^E272K^* mRNA both rescued the *albino* phenotype, while *slc45a2^L374F^* and the double mutant mRNA *slc45a2^E272K/L374F^* failed to rescue (Figure 2). In consideration of Graf et al.’s [12] suggestion that *E272K* in Europeans may be associated with darker skin color, we note that *E272K* does not enhance rescue over that seen using wild-type mRNA (Fig. 2D). There is also no evidence of function in the double mutant construct, indicating that *E272K* does not compensate for the loss of function caused by *L374F* in zebrafish (Fig. 2F). These results confirm the importance of amino acid 374, and suggest that the change at amino acid 272 has little or no effect on pigmentation.

In humans, the predominant allele of *SLC45A2* in Europeans, *L374F,* is clearly not null as it does not cause OCA4; indeed, additional mutations are necessary on this genetic background to cause oculocutaneous albinism type 4 (*OCA4*) [10]. On this basis, it would not have been surprising to find partial rescue by the *L374F* allele. However, no rescue by *L374F* mRNA was observed, even when the amount of mRNA injected was increased until embryonic toxicity (data not shown). These results are consistent with a model in which *L374F* has a lower activity than *L374*, and that a higher dose of this defective protein cannot make up for its deficient function. This result is not unexpected, in light of the high sequence conservation at and around 374 (Figure S5B). At this time, we do not know whether or not adult zebrafish homozygous for *L374F* would be *albino*.

### Assessing the Effect of the *E272K* Allele of *SLC45A2* on Human Skin Color

**Figure 3:**
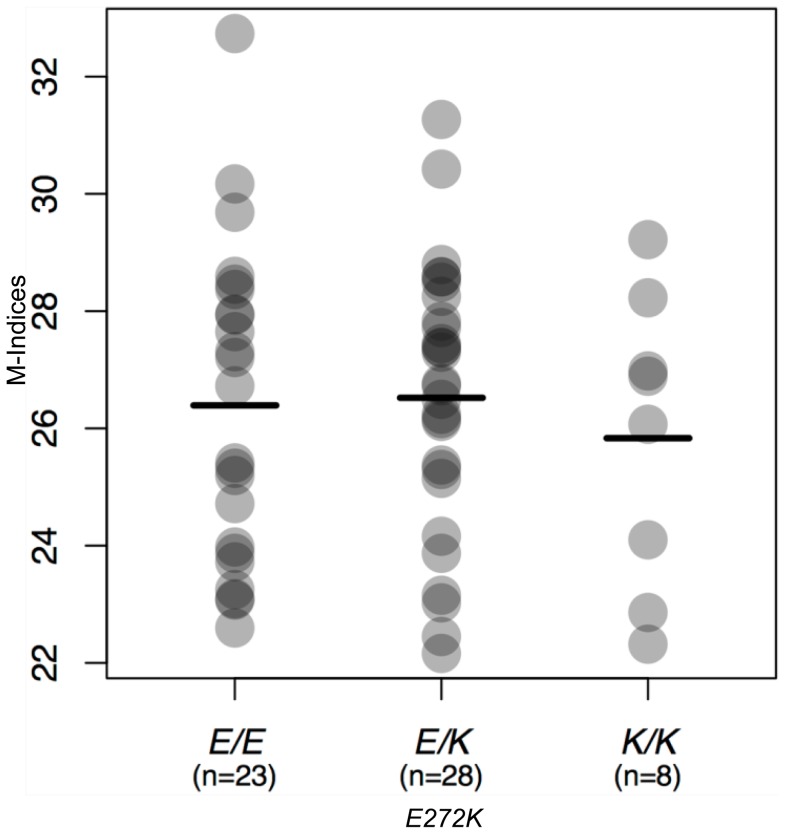
Dot plot showing no significant correlation of *E272K* mutation to the Melanin Index in East Asian populations. Average melanin indices for E/E, E/K and K/K genotypes are 26.4, 26.5, and 25.8, respectively. No significant deviation from Hardy-Weinberg equilibrium was identified for *E272K* SNP in populations of East Asian ancestry (N = 59). Observed and expected genotype frequencies E/E = 23(23.2); E/K = 28(27.6); K/K = 8(8.2). E and K allele frequencies were 0.63 and 0.37, respectively.

We know from prior work that the *L374F* allele of *SLC45A2* has a phenotypic effect in Europeans [6], [7]. In contrast, the *E272K* allele at *rs26722* is of uncertain effect. This allele is present in East Asians at a frequency of ∼0.4. A weak statistical association between this derived allele and darker skin color was initially suggested [12], but later ascribed to linkage disequilibrium [17]. In order to evaluate the specific effect of the *E272K* polymorphism in humans, we genotyped *rs26722* in 59 individuals of East Asian ancestry, with lack of admixture in the last three generations confirmed by questionnaire, and measured their skin color by reflectance spectroscopy [26]. In this sample the *E272K* frequency was 37.3%. All individuals in this sample carried the ancestral allele at position 374, which is consistent with reported ancestry. No statistically significant association was found between skin color measurements and the *E272K* genotype (p = 0.72) (Figure 3); 95% confidence limits for the effect size, assuming an additive model, are −1.12 and +0.77 melanin index units, which is within the technical reproducibility of the assay on an artificial surface. Increasing sample number may increase the sensitivity of this test, but these results rule out a major contribution of *E272K* to East Asian skin color. This result is consistent with the poor amino acid conservation at and around amino acid 272 (Figure S5A).

### Testing a Humanized Zebrafish *A111T* Allele of *SLC24A5* in *golden*

Our original attempts to rescue *golden* with the ancestral human allele in mRNA experiments were associated with severe disruption of embryogenesis with lethality [7], possibly due to effects of ectopic expression. Rescue with the ancestral human allele was successful using cDNA rather than mRNA injection [7], possibly due to fewer toxic effects associated with delayed transcription from cDNA rather than mRNA. In contrast, the *A111T* human allele was able to rescue *golden,* perhaps due to weaker toxic effects associated with the hypomorphic *A111T* allele. These results motivated pursuit of an experimental system that would allow comparative testing with the ancestral allele of human *SLC24A5*.

**Figure 4:**
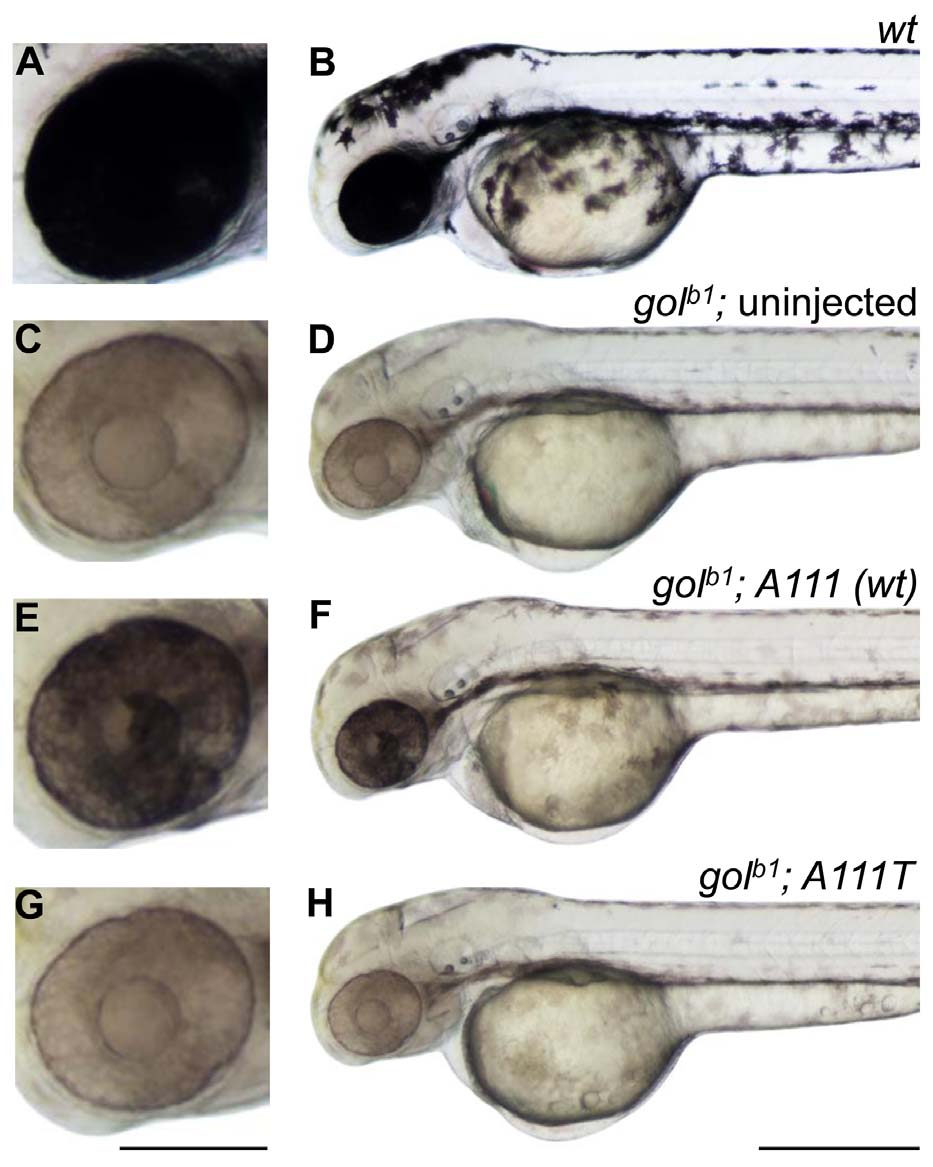
Effect of a human coding polymorphism on zebrafish mRNA rescue of the *golden* phenotype. Lateral views of 48-hpf (A and B) *wt* zebrafish larva (C and D) *gol^b1^* zebrafish larva (E and F) *gol^b1^* larva injected with full-length zebrafish *slc24a5 (wt) mRNA* (500 pg) and (G and H) *gol^b1^* larva injected with full-length zebrafish *slc24a5 mRNA* with a single nucleotide change (500 pg), coding for the orthologous human derived *A111T* allele. Scale bars in (A, C, E, G) 150 µm, (B, D, F, H) 400 µm.

Constructs containing the *wt slc24a5* cDNA were engineered to carry the *A111T* variant, and rescue experiments were performed in *golden^b1^* embryos. Injection of *slc24a5^wt^* but not *slc24a5^A111T^* mRNA rescued the pigmentation phenotype by 48 hours post-fertilization (hpf) (Figure 4). This result is consistent with observations that the human alleles exhibit transport differences *in vitro* [27]. Our previously reported partial rescue of the *golden* phenotype by human alleles of *SLC24A5* was assayed at 60–72 hpf [7]. Here, we scored pigmentation at 48 hpf, which is a more stringent test of rescue than the later time point.

### Amino Acid Sequence Conservation in OCA Genes of Human and Zebrafish

**Figure 5:**
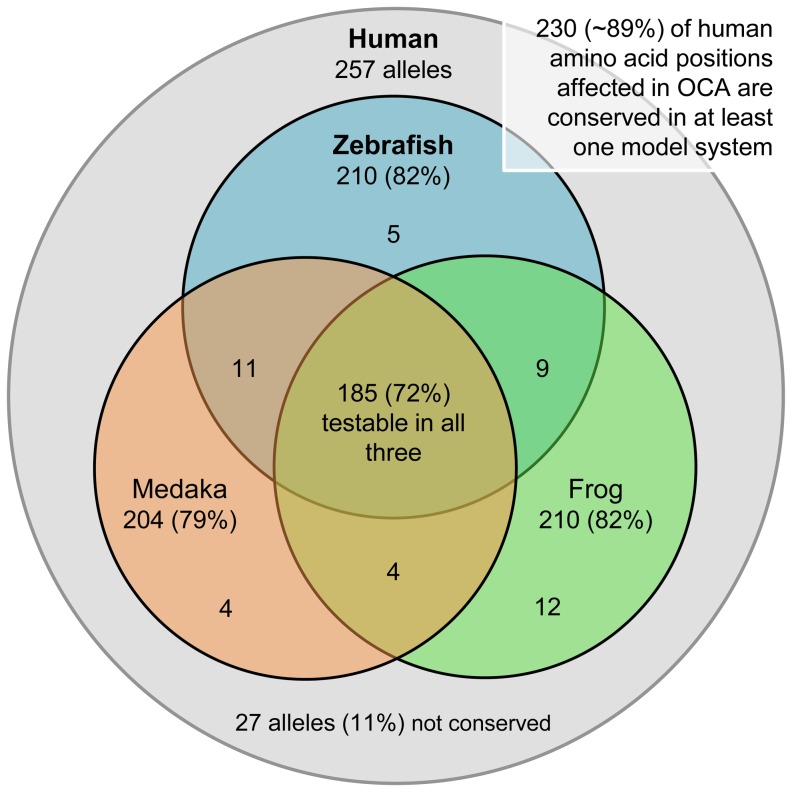
Phylogenetic conservation of amino acid changes associated with OCA. The Venn diagram illustrates the number of mutations changed across all four OCA genes (*TYR, OCA2, TYRP1,* and *SLC45A2*) that can be tested in zebrafish, western clawed frog, and/or medaka on account of wild-type amino acid conservation. Since different mutations can affect different nucleotides in a single codon and different mutations at the same nucleotide can result in different amino acids, the 257 alleles are found in 222 loci.

The potential applicability of HuZOR to other human phenotypes may be estimated from the proportion of clinically important human mutations that affect conserved coding sequence. Since albinism is easily recognized as a human phenotype, we asked what proportion of phenotypically important mutations affects coding sequences that are evolutionarily conserved for the four known human oculocutaneous albinism (OCA) syndromes. About 2/3 of OCA phenotypes map to the four known OCA genes (M. Brilliant, pers. commun.). Since the unmapped mutations causing OCA phenotypes may also affect coding sequence, at least 2/3 of OCA syndromes are caused by changes in known coding sequence. In a survey of characterized OCA mutations (OMIM and U Minnesota), we found 257 missense mutations in *TYR, OCA2, TYRP1,* and *SLC45A2* that are implicated in the four corresponding forms of OCA: OCA1, OCA2, OCA3, and OCA4. We asked, for each position, whether the corresponding amino acid is conserved in zebrafish. Overall, 210, or 82% of these loci were conserved (Figure 5).

## Discussion

It is difficult in human-derived tissues to distinguish the effects of a single nucleotide change in DNA sequence from the effects of other uncontrolled variation (outside and/or inside the gene in question; see for example, [28]). In HuZOR, we are focusing on DNA sequence changes that result in a change in amino acid. In this approach, mutations outside of the gene are not a factor, and furthermore, as shown above for the two coding SNPs in *SLC45A2*, specific combinations of sequence variations within a single gene can be tested (panel F of Figure 2). The ability to test the effect of a single amino acid change or combination of changes is a key strength of HuZOR.

**Figure 6:**
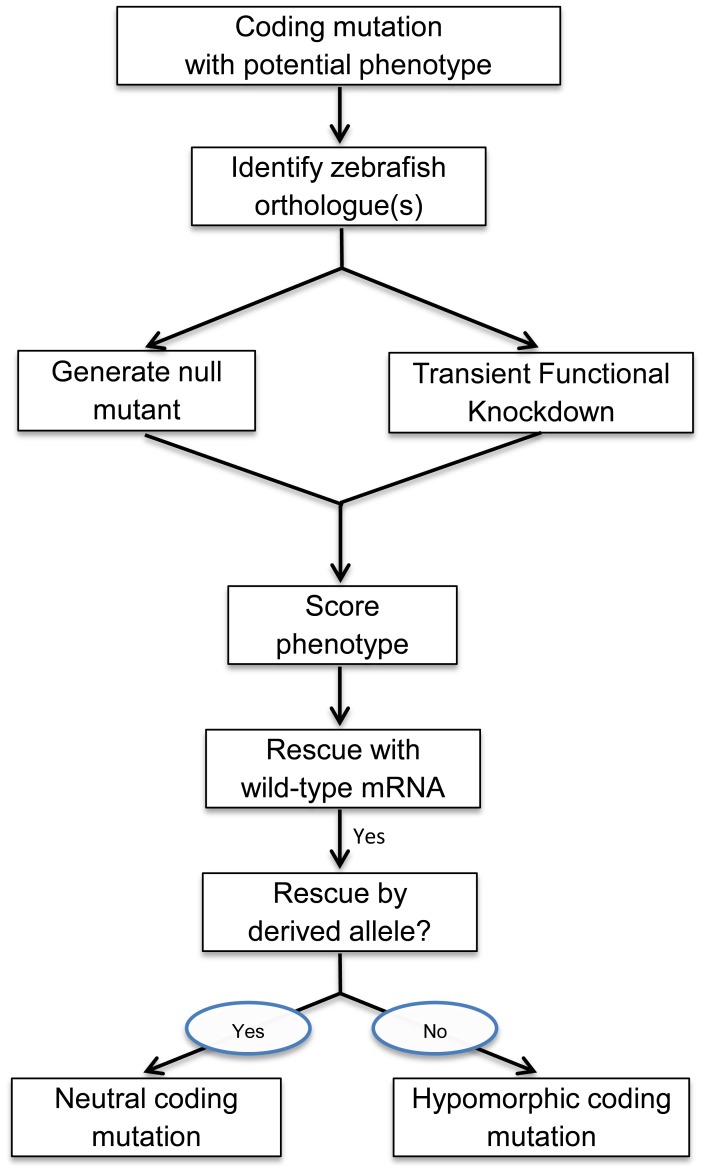
Flow chart for testing coding mutations based on the HuZOR approach. Candidate functional coding mutations are first identified from genome sequences. After an orthologue (or potentially a pair of orthologues) is identified, null mutants and/or gene-specific transient functional knockdown embryos are generated with morpholinos or TALENs in zebrafish or other model species. Phenotypes are then scored. Rescue of mutant or functional knockdown phenotypes are then tested by microinjection of wild-type mRNA (and potentially cDNA). If rescue is successful, and if the corresponding amino acid is conserved, zebrafish mRNA containing the orthologous amino acid change is then generated, and ability of the derived mRNA to rescue the mutant phenotype is then tested. Phenotypic rescue by the derived mRNA is evidence in support of the original variant being phenotypically neutral in humans. In contrast, loss of the ability to rescue as a result of an orthologous amino acid change suggests a deleterious effect on protein function. Mutations in regulatory elements, such as a mutation disrupting a phosphorylation site, may potentially have hypomorphic or hypermorphic effects.

As an estimate of the proportion of phenotypically important coding mutations that are conserved in zebrafish, we showed above that ∼82% of human coding mutations linked to OCA phenotypes are conserved in zebrafish. Since mutants and functional knockdowns may be generated and mRNA rescue can be done in two other small vertebrate models, the western clawed frog (*Xenopus tropicalis*) [29], and medaka (*Oryzias latipes*) [30], we also asked whether the changed amino acids in human OCA are conserved in those two species (Figure 5). The percentages of conservation of amino acid at those positions for Xenopus and medaka were similar to zebrafish: 82% and 79%, respectively. If all three species could be used, then 90% of coding mutations in human OCA could be tested in at least one of the three model systems. This level of evolutionary conservation is consistent with the functional importance of the particular amino acids associated clinically with OCA, and suggests that the HuZOR approach (Figure 6) may be potentially extended to other small vertebrate species such as *Xenopus tropicalis* and medaka.

Some disease susceptibility alleles have been predicted to be the result of mutations causing mild, rather than null phenotypes [31]. It is therefore useful that the zebrafish-based HuZOR approach can be sensitive enough to detect not only null alleles (common in recessive Mendelian traits of strong effect), but also mildly hypomorphic human alleles such as those tested here. The ability to detect mild phenotypes will be dependent upon the ability to detect a range of mutant phenotypes, variability in mutant phenotype and variability of mRNA rescue. Given that many human disease susceptibility phenotypes are dependent upon environmental factors, it will also be desirable, when possible, to test environmental manipulations within the context of HuZOR. The extent to which HuZOR can be applied to phenotypes beyond pigmentation remains to be determined, and will depend upon the sensitivity of phenotypic assays for detecting mutant phenotypes in orthologous zebrafish mutants or transient knock-downs, and the ease of mRNA-based rescue.

The potential of HuZOR will grow with the addition of quantitative analysis and more comprehensive, higher resolution imaging. Pigmentation is cell-specific and rescue is variable on a cell-to-cell basis, making quantitation currently challenging. Detection of morphological phenotypes depends upon the resolution of the imaging technique used. Many phenotypes escape detection using the most common visual tool for studying zebrafish embryos: the dissecting stereomicroscope [32] [G. Thomas, B. Canada, and K. Cheng, unpublished]. Both quantitation and sensitivity issues may be addressable in the future by improvements to 3D whole-animal imaging methods with cell resolution, such as histology or microCT [32].

Several other aspects of HuZOR deserve note. Our rescue experiments with human *SLC24A5* mRNA illustrate how toxicity, such as that caused by ectopic expression of the gene in question, can obscure rescue. In such cases, injection of DNA constructs yielding tissue-specific expression can be considered as an alternative to mRNA injection. Rescue might potentially be limited when the mRNA is large, which can potentially result in more limited distribution of the mRNA throughout the embryo. When rescue of lethal phenotypes is being tested, mutants can be generated from heterozygotes, but only ¼ of embryos will be mutant in crosses between heterozygotes. In such a setting, injected fish can be genotyped for homozygosity of the mutation subsequent to injection. HuZOR as described is limited by the time within which mRNA rescue exerts its impact and the age at which a mutant phenotype is observed. Phenotypes that only appear in older animals may require rescue of mutant phenotypes with transgenic constructs.

HuZOR may not be possible for genes lacking zebrafish mutant phenotypes, unless an environmental manipulation can yield a phenotype. For human genes lacking orthologues in zebrafish, assays in a model system with a human orthologue will be needed. For some genes, the model organism will have two paralogues that are orthologous to a human gene [33]. In these cases, the use of HuZOR may be limited by the extent to which the potentially affected human functional domain is represented on one or both zebrafish paralogues. In this setting, HuZOR may still be possible using either mutant alone, or a double mutant if both orthologues are functional.

The necessity of acquiring a mutant or transient gene knockdown to use HuZOR is becoming less of a barrier as increasingly sophisticated tools for targeting genes in zebrafish are developed. Mutants are being generated using an ENU-mutagenesis-based method, TILLING [34], zinc finger nucleases (ZFNs) [5], and more recently, TALENs [4], which are proving to be efficacious in zebrafish [35]. Gene knockdowns using morpholinos is established [3], and with slight increases in efficiency, it may become possible to mutate a sufficient proportion of somatic cells to efficiently generate targeted knockouts by microinjections of fertilized eggs [35].

It has yet to be determined how frequently the physiology is adequately conserved to allow testing for phenotypes that are directly applicable to humans. However, advantage can be taken of the fact that many mutant phenotypes are pleiotropic, and cross organ systems [24]. Any one of multiple phenotypes, can potentially be used as surrogates for testing gene function by rescue. These other phenotypes may not only be useful for HuZOR in instances where the human gene function is unknown; they may be informative about gene function.

The ease of generating embryos, embryonic transparency and rapid development make the zebrafish an economical choice of vertebrate model organism for assessing the potentially phenotypic effects of specific human coding mutations. Characterization of zebrafish mutant phenotypes can also be based on changes in patterns of gene expression as assayed by in situ hybridization [36], transgene fluorescence [37], and microanatomy (as studied by histology [38] or microCT [32]).

Candidate coding mutations are found by DNA sequence analysis of genomic regions mapped to potential human disease alleles and as byproducts of the sequencing of individual genomes. We have described here a potentially generalizable approach to assessing the effect of coding mutations *in vivo* in a vertebrate model system (Figure 6). The method requires identification of an orthologous gene, generating a mutant or transient functional knockdown, finding a mutant phenotype to follow, and testing mRNA constructs containing individual orthologous coding mutations for their ability to rescue the mutant phenotype. It will be important to determine the extent to which this approach can be applied to genes associated with phenotypes other than pigmentation. For example, 392 coding-region SNPs were found in a genome-wide survey relevant to cardiovascular, endocrine and neuropsychiatric phenotypes [39], but experimental evaluation of these SNPs is lacking. The number of genes that can potentially be tested using this approach is growing as new mutants are generated for the zebrafish phenome project. It is worth noting that coding mutations in other vertebrate organisms may also be testable using this approach. The further development of assays such as HuZOR for testing the functional significance of specific human coding mutations may contribute to the utility of individual human genomes in personalized medicine.

## Materials and Methods

### Fish Culture

All research involving zebrafish was approved by the Penn State College of Medicine Institutional Animal Care and Use Committee (2004–102). Wild-type zebrafish were purchased from Lyles Tropical Fish (Ruskin, FL); *gol^b1^* and *alb^b4^* mutants were purchased from the Zebrafish International Resource Center (Eugene, OR). Koichi Kawakami’s Lab supplied *alb^nk1^*, and Stephen Johnson’s lab supplied *alb^b4→SJ1^*. The *alb^b4→SJ1^* allele was historically derived from the *alb^b4^* allele from University of Oregon [40]. Embryos were maintained at 28.5°C as described previously [41].

### Cell Culture

Malignant melanoma cell lines, MNT-1 of European origin and HM3KO of Korean origin [42] (from Dr. Yoko Funasaka through Dr. Hee-Young Park), were grown to generate the derived and ancestral alleles of human *SLC45A2* mRNA, respectively, and maintained in Dulbecco’s Modified Eagle’s Medium (DMEM) and supplemented with 10% fetal bovine serum (FBS), 100 unit/mL penicillin, and 100 ug/mL streptomycin at 37°C, 5% CO_2_. MNT-1 media also contained 7.5% sodium bicarbonate and 200 mM L-glutamine.

### gDNA and Total RNA Isolation from Zebrafish and Human Cell Culture, cDNA Cloning

For gDNA extraction from pooled embryos or fin clips, we used a DNeasy Blood and Tissue Kit (Qiagen). The PCR products were sequenced in the Penn State College of Medicine Macromolecular Core Facility. Total RNAs were isolated from human cell lines as well as *wt*, *gol^b1^*, *alb^nk1^* and *alb^b4→SJ1^* zebrafish larvae (∼48 hpf) using the RNeasy kit (Qiagen). 1 µg of total RNA was reverse-transcribed using the M-MLV Reverse Transcriptase (Invitrogen). Primers, including restriction sites for further subcloning, were designed for full-length zebrafish and human *slc45a2,* as well as zebrafish *slc24a5* cDNA amplification (Table S1). PCR products were cloned using the TOPO TA Cloning system and pCR®II-TOPO vector.

### Zebrafish Knockdown and Rescue Experiments

5′UTR and start site translational fluorescent-tagged morpholino oligonucleotides (Gene Tools) were used for *slc45a2* and *slc24a5* knockdown in zebrafish:

1. *slc45a2_*5′UTR, 5′-TCTTGATTCCTAGTGCATAGTTGAG-3′
2. *slc45a2_*ATG, 5′-GCTGGTCCTCAGTAAGAAGAGTCAT-3′
3. *slc24a5_*5′UTR, 5′-CATTCAGCAGAACACAGATGACGGA-3′

Morpholinos were prepared in 1x Danieau buffer containing 0.05% Phenol Red, and were injected into 1–2 cell stage zebrafish embryos. Morpholino injections were verified using a fluorescent microscope.

Zebrafish *slc45a2* allele cDNAs contained: 1) *Glu272Lys* with *Leu374* (derived for 272 and ancestral for 374); 2) *Glu272* with *Leu374Phe* (ancestral for 272 and derived for 374); and 3) *Glu272Lys* with *Leu374Phe* (derived for both alleles). All three were generated from zebrafish *wt* cDNA - *Glu272* with *Leu374* (ancestral for both alleles) using PCR mutagenesis. Zebrafish-derived *Ala111Thr slc24a5* allele cDNA was produced in the same way, from zebrafish *wt* cDNA: an orthologue of the human ancestral allele, *Ala111*.

For human rescue experiments, *SLC45A2* cDNA was amplified using RNA from human MNT-1 (European origin) and HM3KO (Asian origin) cell lines as templates. The internal primers as well as the forward and reverse primers for PCR mutagenesis are shown in Table S1.

All cDNAs were subcloned from the pCR®II-TOPO vector into the pT3TS vector. Capped mRNA transcripts were generated *in vitro* from the linearized plasmids using mMESSAGEmMACHINE T3 kit (Ambion) according to the manufacturer’s protocol. The integrity of the RNA was verified by agarose gel electrophoresis. RNA rescue experiments were performed by injecting 100–1400 pg RNA into *albino* and *golden* embryos at 1–2 cell stage.

### Zebrafish Embryo Imaging

Photography was done in the Penn State Zebrafish Functional Genomics Core Facility. Fluorescent embryos were observed with an OLYMPUS MVX10 microscope and images of zebrafish embryos were acquired using a LEICA MZFL III microscope and QCapture software, equipped with ROLERA-MGi (QIMAGING) and RETIGA 4000R (QIMAGING) digital cameras, respectively.

### East Asian DNA Collection and Genotyping

This research is approved by the Penn State College of Medicine Institutional Review Board (29269EP). We collected saliva samples (N = 59) from people of East Asian ancestry from Hershey, PA USA. A set of simple questions was asked to ensure that participants were of East Asian ancestry (Chinese, Taiwanese, Korean, Japanese, Vietnamese, and Malaysian).

Saliva samples were collected using DNA self-collection kit OG-500 (Oragene), and DNA was extracted using Oragene prepIT.2LP (DNA Genotek) protocol. The saliva was incubated at 50°C overnight to increase the DNA yield and ensure inactivation of all nucleases. DNA quality was measured using Qubit Fluorometer (Invitrogen).

PCR was performed and genotypes were defined by sequencing for *A111T* (*SLC24A5*) and *L374F* (*SLC45A2*) SNPs to verify that all East Asian samples were free from European admixture. The same process was performed for *E272K* (*SLC45A2*) to see if there was any correlation between genotype and pigmentation measurement.

### Melanin Measurement using Reflectometry

The melanin contribution can be quantitatively measured by reflectance spectrophotometry, using the L* value from the Commission International d’Eclairage (CIE) L*a*b* color system [43]. We measured constitutive skin pigmentation for East Asian individuals on the upper inner arm to minimize the potentially confounding effects of sun exposure. We used a Datacolor CHECK^PLUS^ spectrophotometer for our measurements, and report the calculated melanin index (M). Each reported M index is an average of three readings and calculated based on the following equation: M = 100 log_10_ (100/% reflectance) [26]. All subjects were sampled and measured in December 2011. Before measuring, the instrument was calibrated using black and white calibration standards provided by the manufacturer. Care was taken not to apply too much pressure to the skin with the spectrophotometer, since doing so could occlude blood from the region being measured [44].

### Statistical Analysis

M-indices, calculated from reflectance [26], were grouped by *E272K* allele genotype and graphed in Figure 3 as a column scatter graph with alpha blending. Using an additive genetic model, or trend test, a linear regression analysis did not identify a statistically significant association of *E272K* allele number on skin pigmentation in this East Asian population (p = 0.72). Statistical calculations were performed using R version 2.15.0. Performing a post-hoc power calculation using the G*Power program version 3.1, a total sample size of 59 subjects is sufficiently powered to detect a statistically significant association yielding 97% power at a.05 alpha level in detecting a moderate effect size given by a Cohen’s *f* ^2^ effect size of.25.

### DNA Sequences

*Homo sapiens SLC45A2* (NCBI Reference Sequence: NM_016180.3); *Mus musculus SLC45A2* (NM_053077.3); *Oryzias latipes slc45a2* (NM_001104758.1); *Xenopus laevis* (NM_001095910.1); *Danio rerio slc45a2* (NM_001110377.1); *Homo sapiens SLC24A5* (NM_205850.2); *Danio rerio slc24a5* (NM_001030280.1).

### Protein Sequences

*Danio rerio tyr* (NCBI GenBank: AAN17339.1); *Oryzias latipes tyr* (BAA06155.1); *Xenopus tropicalis tyr* (NP_001096518.1); *Danio rerio oca2* (XP_695807.5); *Oryzias latipes oca2* (NP_001098262.1); *Xenopus tropicalis oca2* (XP_002937026.1); *Danio rerio tyrp1(b)* (AAH76406.1); *Oryzias latipes tyrp1* (Ensembl ENSORLP00000005420); Xenopus tropicalis *tyrp1* (NP_001016476.1); *Danio rerio slc45a2* (AAI54628.1); *Oryzias latipes slc45a2* (P58355.1); *Xenopus tropicalis slc45a2* (NP_001011335.1).

## Supporting Information

Figure S1 **Injection of human mRNAs fail to rescue** ***albino*** **.** (A) Wild-type zebrafish *slc45a2* mRNA (1400 pg) injected into *albino* embryos rescues pigmentation (arrows) while *SLC45A2* mRNA of human (B) ancestral (*L374)* (1400 pg) and (C) derived (*L374F)* (1400 pg) alleles do not. Scale bar 300 µm.
(TIF)

Figure S2 **Morpholino knockdown of** ***slc45a2*** **phenocopies** ***albino*** **.** Lateral views of wild-type 48-hpf zebrafish larvae that are uninjected (A) or injected (B) with 8 ng morpholino targeted to the 5′UTR of *slc45a2*. Scale bar 200 µm.
(TIF)

Figure S3 **Co-injection of morpholino and mRNA for** ***slc45a2*** **into the zebrafish embryos causes substantial developmental defects.** (A) zebrafish wild-type embryos (B) Injection of zebrafish embryos with 5′UTR morpholino (8 ng) against *slc45a2* (non-overlapping with the mRNA sequence) reduces pigmentation, while (C) coinjection with *slc45a2* mRNA (500 pg) causes severe developmental defects that interfere with detection of phenotypic rescue. Scale bar 600 µm.
(TIF)

Figure S4 **Co-injection of morpholino and mRNA for** ***slc24a5*** **into the zebrafish embryos causes substantial developmental defects.** (A) zebrafish *wt* embryos (B) Injection of zebrafish embryos with 5′UTR morpholino against *slc24a5* (non-overlapping with the mRNA sequence) reduces pigmentation and, (C) and, with coinjection with *slc24a5* mRNA (500 pg) causes severe developmental defects similar to those seen in Figure S3, panel C, precluding the detection of rescue. Scale bar 300 µm.
(TIF)

Figure S5 **Amino acid alignment of** ***slc45a2*** **from various vertebrate species** showing that the *E272* region (A) is not conserved as well as the *L374* region (B).
(TIF)

Table S1 **Primers used for study.**
(XLS)
