# Supplementary figures and images for: Functional Assessment of Human Coding Mutations Affecting Skin Pigmentation Using Zebrafish
Source: PLoS One. 2012 Oct 10;7(10):e47398. doi: 10.1371/journal.pone.0047398 (PMC3468441; doi:10.1371/journal.pone.0047398)

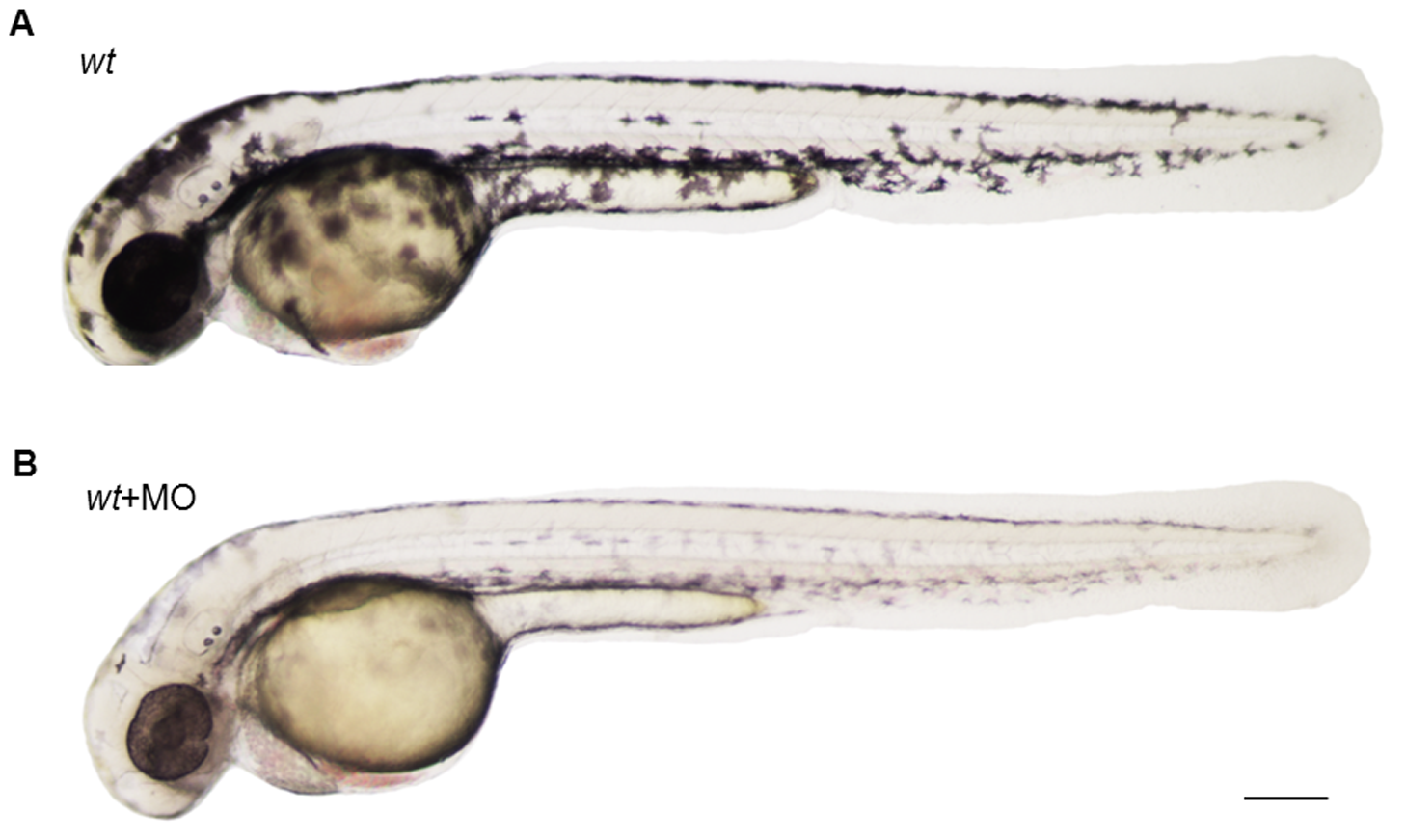

Supplement: Figure S1 — Injection of human mRNAs fail to rescue albino . (A) Wild-type zebrafish slc45a2 mRNA (1400 pg) injected into albino embryos rescues pigmentation (arrows) while SLC45A2 mRNA of human (B) ancestral (L374) (1400 pg) and (C) derived (L374F) (1400 pg) alleles do not. Scale bar 300 µm. (TIF) [file pone.0047398.s001.tif]

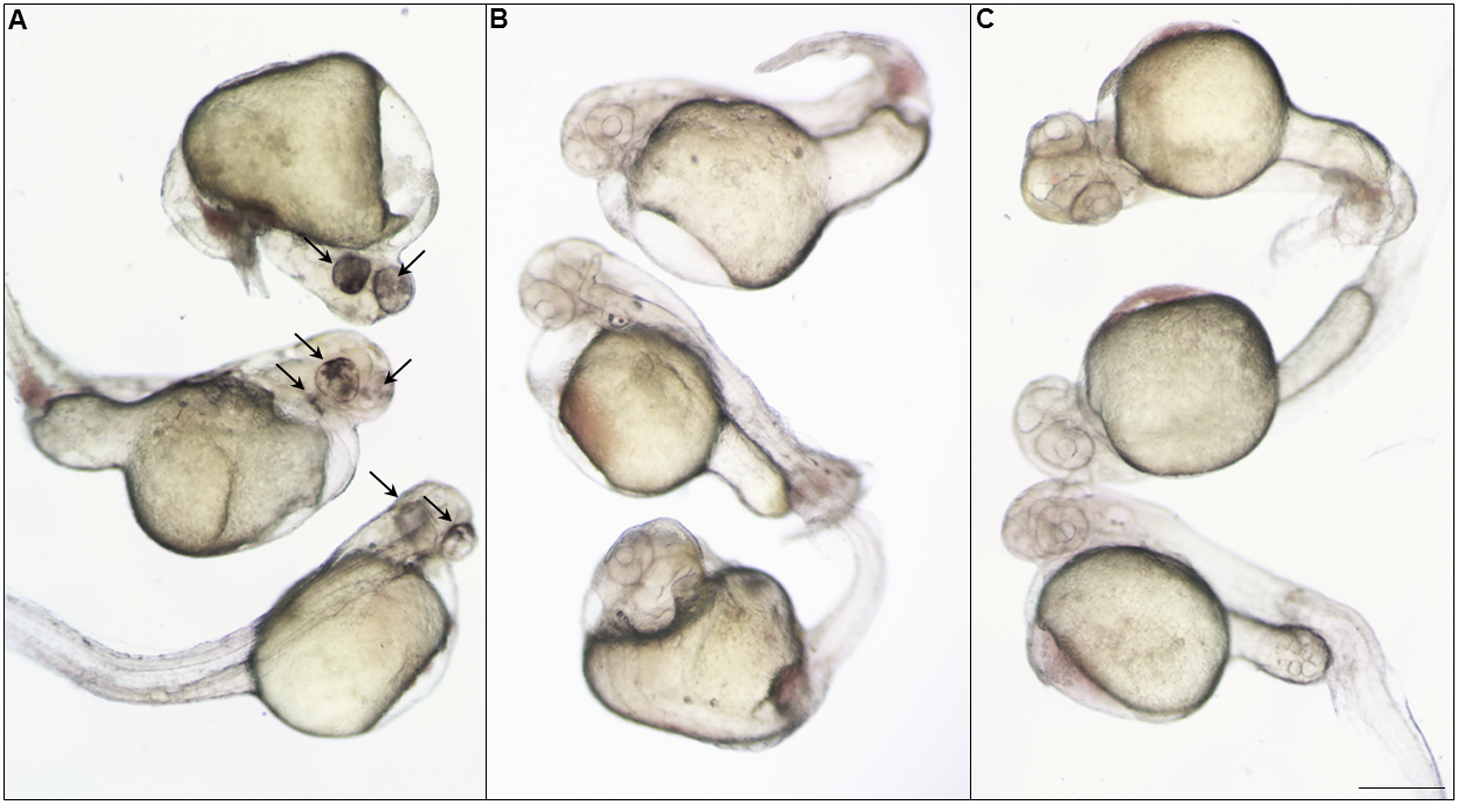

Supplement: Figure S2 — Morpholino knockdown of slc45a2 phenocopies albino . Lateral views of wild-type 48-hpf zebrafish larvae that are uninjected (A) or injected (B) with 8 ng morpholino targeted to the 5′UTR of slc45a2. Scale bar 200 µm. (TIF) [file pone.0047398.s002.tif]

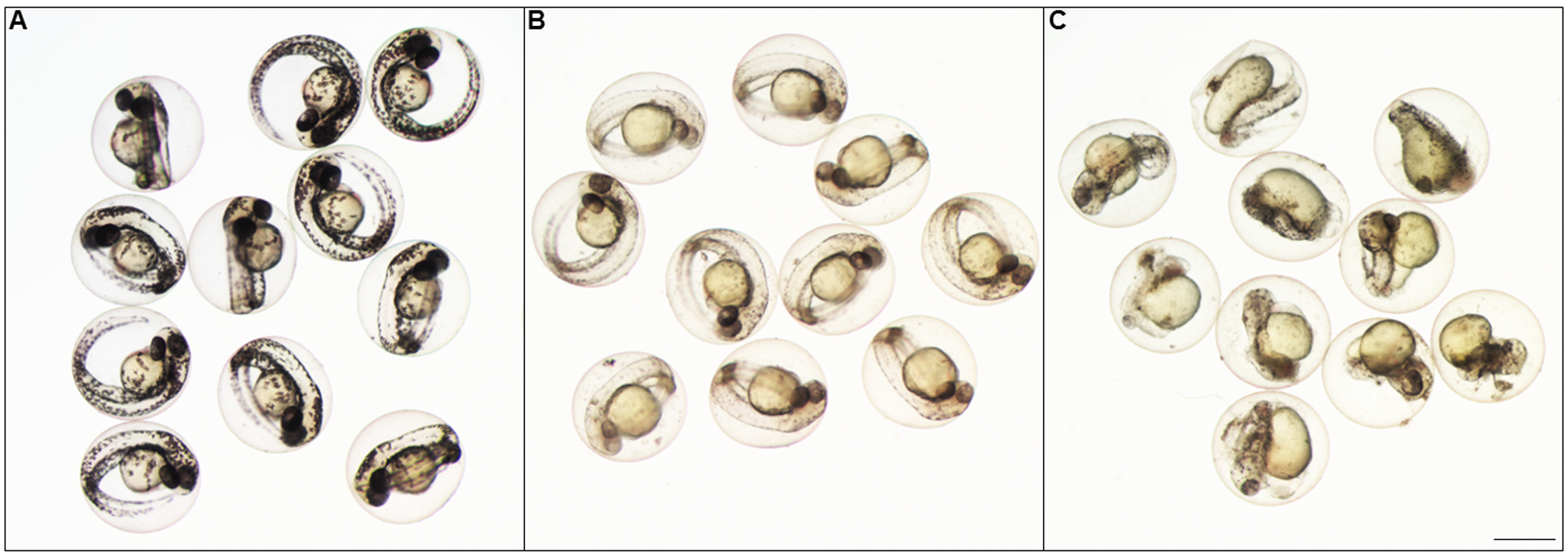

Supplement: Figure S3 — Co-injection of morpholino and mRNA for slc45a2 into the zebrafish embryos causes substantial developmental defects. (A) zebrafish wild-type embryos (B) Injection of zebrafish embryos with 5′UTR morpholino (8 ng) against slc45a2 (non-overlapping with the mRNA sequence) reduces pigmentation, while (C) coinjection with slc45a2 mRNA (500 pg) causes severe developmental defects that interfere with detection of phenotypic rescue. Scale bar 600 µm. (TIF) [file pone.0047398.s003.tif]

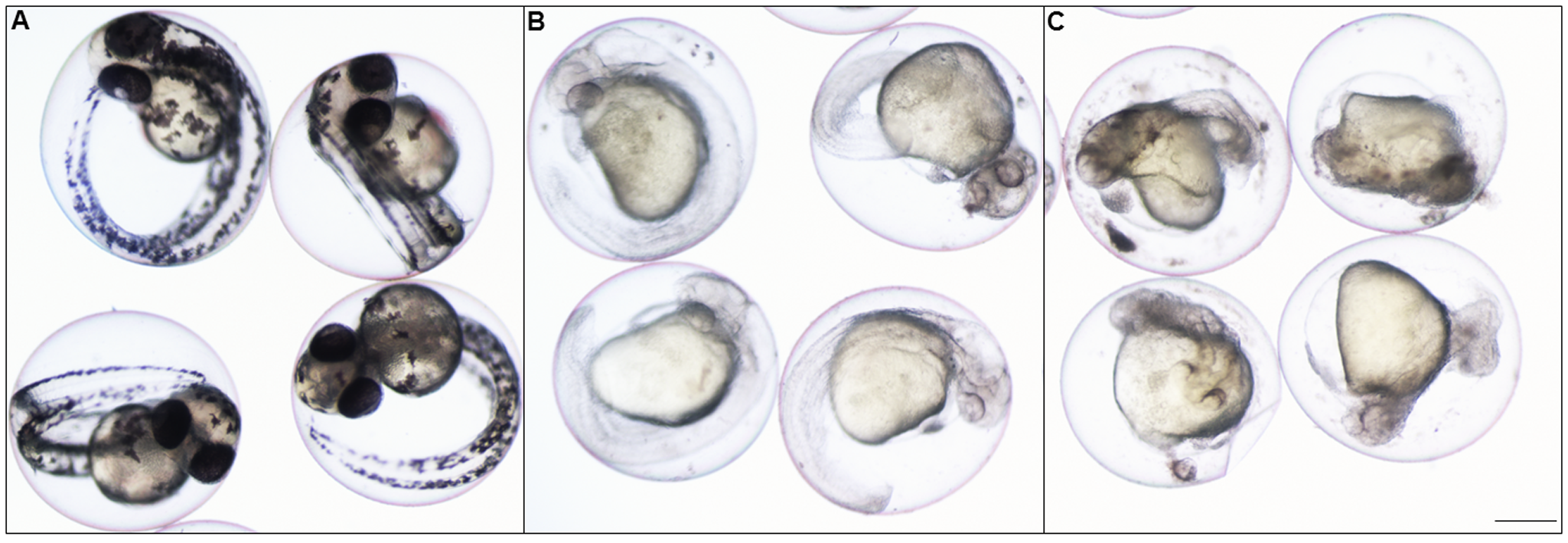

Supplement: Figure S4 — Co-injection of morpholino and mRNA for slc24a5 into the zebrafish embryos causes substantial developmental defects. (A) zebrafish wt embryos (B) Injection of zebrafish embryos with 5′UTR morpholino against slc24a5 (non-overlapping with the mRNA sequence) reduces pigmentation and, (C) and, with coinjection with slc24a5 mRNA (500 pg) causes severe developmental defects similar to those seen in Figure S3, panel C, precluding the detection of rescue. Scale bar 300 µm. (TIF) [file pone.0047398.s004.tif]

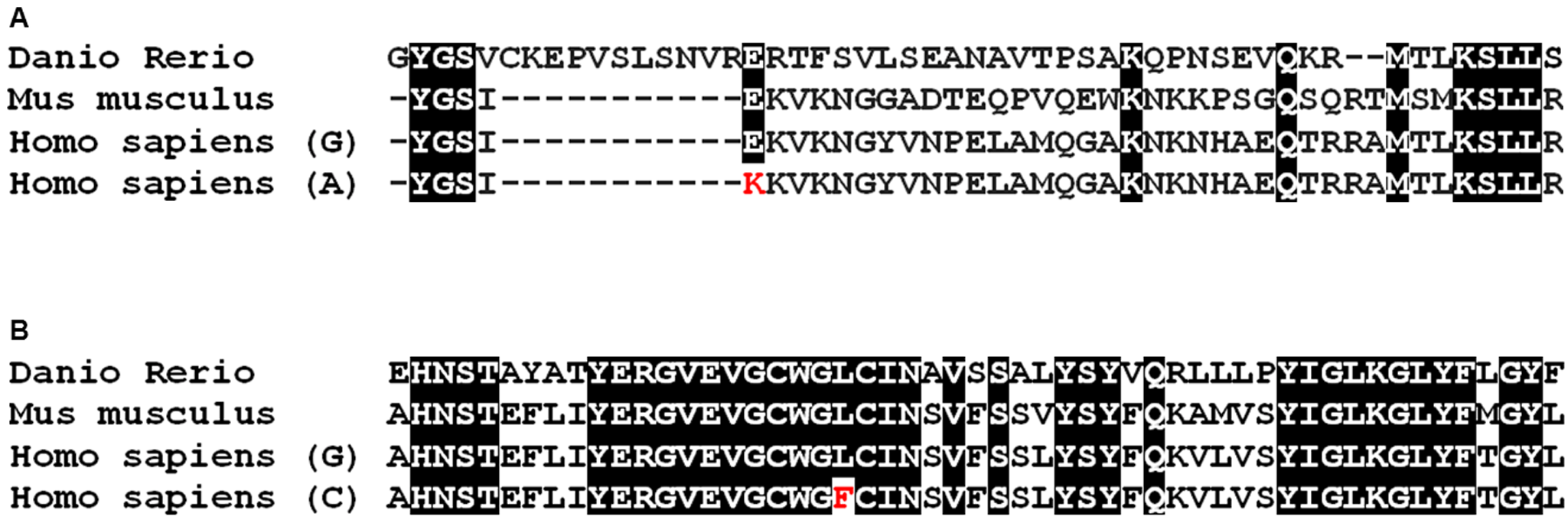

Supplement: Figure S5 — Amino acid alignment of slc45a2 from various vertebrate species showing that the E272 region (A) is not conserved as well as the L374 region (B). (TIF) [file pone.0047398.s005.tif]
